# Investigating Pathogenetic Mechanisms of Alzheimer’s Disease by Systems Biology Approaches for Drug Discovery

**DOI:** 10.3390/ijms222011280

**Published:** 2021-10-19

**Authors:** Shan-Ju Yeh, Ming-Hsun Chung, Bor-Sen Chen

**Affiliations:** Laboratory of Automatic Control, Signal Processing and Systems Biology, Department of Electrical Engineering, National Tsing Hua University, Hsinchu 30013, Taiwan; m793281@gmail.com (S.-J.Y.); gnshiun@gmail.com (M.-H.C.)

**Keywords:** Alzheimer’s disease, genetic and epigenetic networks, systems biology, drug discovery

## Abstract

Alzheimer’s disease (AD) is the most common cause of dementia, characterized by progressive cognitive decline and neurodegenerative disorder. Abnormal aggregations of intracellular neurofibrillary tangles (NFTs) and unusual accumulations of extracellular amyloid-β (Aβ) peptides are two important pathological features in AD brains. However, in spite of large-scale clinical studies and computational simulations, the molecular mechanisms of AD development and progression are still unclear. In this study, we divided all of the samples into two groups: early stage (Braak score I–III) and later stage (Braak score IV–VI). By big database mining, the candidate genetic and epigenetic networks (GEN) have been constructed. In order to find out the real GENs for two stages of AD, we performed systems identification and system order detection scheme to prune false positives with the help of corresponding microarray data. Applying the principal network projection (PNP) method, core GENs were extracted from real GENs based on the projection values. By the annotation of KEGG pathway, we could obtain core pathways from core GENs and investigate pathogenetic mechanisms for the early and later stage of AD, respectively. Consequently, according to pathogenetic mechanisms, several potential biomarkers are identified as drug targets for multiple-molecule drug design in the treatment of AD.

## 1. Introduction

Alzheimer’s disease (AD) is a brain disorder of neurodegeneration. The symptoms of AD are characterized into three main groups. The first group is described as cognitive dysfunction including language difficulties, memory loss and mental incapacity. The second group consists of psychiatric symptoms, such as hallucinations, delusions, depression and agitation. The third group comprises inability to perform activities of daily living, for example, driving, cooking and shopping [1]. Clinically, we can observe that most of early-stage AD patients reveal losing spatial memory, long-term memory and short-term memory. Meanwhile, they tend to be very moody and experience emotional extremes in a short period of time. In the later stage of AD, patients have difficulties taking good care by themselves so they become bedridden and cannot do anything until the end of life. This is a result of chronic damage to the brain from the medial temporal lobe structures to the entire neocortex [2]. Epidemiologic studies indicate that from 2006, 26.6 million individuals suffer from AD worldwide and the number of patients may increase fourfold to 106.8 million by the year 2050 [3]. Long-term care of individuals with a severe variety of neuropathology and dementia of AD has undoubtedly grown. It is expected that AD will place significant burden on social welfare and bring huge financial pressure to the family. Not only various experiments of neuroscience have been performed to possibly achieve satisfactory results, but also manpower and material resources have been put into to struggle against neuropathologic changes with cognitive status.

The pathogenic progression of AD contributes to forming irreversible damage of neurons causing cognitive dissonance and progressive dementia. In terms of biochemical perspectives, the disease has two hallmarks. The first one is abnormal aggregations of intracellular neurofibrillary tangles (NFTs). The hyperphosphorylated tau is found in AD. It makes tau detach from microtubules resulting in the formation of soluble tau aggregates and insoluble paired helical filaments that ultimately form NFTs [4]. The second one is unusual accumulations of extracellular amyloid-β (Aβ) peptides. The transmembrane protein, amyloid precursor protein (APP), is sequentially cleaved by two proteases, which are β-secretase (β-site APP cleaving enzyme 1, BACE1) and γ-secretase, to release multiple isoforms of Aβ peptides. The most aggregation prone of Aβ_42_ isoform aggregates tend to form toxic oligomers and deposits in amyloid plaques [5]. Amyloid plaques are relatively specific for AD, whereas tangles are also found in other disorders [6]. Moreover, a staging method for AD has been proposed in 1991 based on NFTs appearing in several brain regions: stages I–II → transentorhinal stages, stages III–IV → limbic stages, and stages V–VI → isocortical stages [7]. Briefly, Braak et al. have distinguished six stages according to multiple regions affected in human brains.

The diagnosis of AD confers a poor prognosis with a mean survival time of approximately 5–8 years from symptom onset [8,9]. The survival time is heterogeneous, and many factors are postulated to influence mortality. Aging, male gender, and worse disease severity have consistently shown to decrease the survival time [10,11,12,13]. The main enzymes participating in pathogenic mechanisms of AD are β-secretase and γ-secretase. Hence, the secretase inhibitors blocking the enzymes that cleave the APP to prevent the formation of insoluble Aβ peptides, which are responsible for the formation of senile plaques, receive significant attention. Regarding the reduction of Aβ peptides, many studies concerning the inhibition of β-secretase have been conducted in an attempt to retard the progression of AD [14,15,16,17]. In addition, Simers et. al. obtained good results in clinical trials of inhibitors of γ-secretase without adverse effects [18]. Furthermore, AD is also associated with the deficit of the neurotransmitter acetylcholine (Ach) and oxidative stress caused by exacerbation of glutamatergic transmission [19,20]. The drugs act as inhibitors of cholinesterase (Acetylcholinesterase—AchE, and Butyrylcholinesterase—BChE), which are enzymes responsible for degradation of the Ach in the synapse. Thus, cholinesterase inhibitors could increase the availability of neurotransmitters in the synapse, reducing the symptoms of AD [21]. For the treatment of AD in mild to severe stages, memantine was licensed by the U.S. Food and Drug Administration (FDA). It could reduce excessive glutamatergic neurotransmission, decrease hyperphosphorylation of tau protein, and protect against toxicity induced by the Aβ peptides [22]. Over the past few years, Aβ immunotherapy has become one of the most exciting areas of research in AD. It began to draw attention after the publication of the first immunization paper, which has shown that amyloid pathology was reduced in an APP transgenic mouse model after vaccination with aggregated Aβ_42_ [23]. The complex pathogenic mechanisms of AD hinder the development of fully effective drugs. Additionally, maintenance of the treatment from the patients is often difficult. The possibility for AD treatment based on the combination of repurposed drugs is worth studying.

Some literatures have demonstrated that genetic elements are touched upon in AD risk factors: APOEε4 [24,25], TREM2 [26,27], CASS4, CELF1, FERMT2, HLA-DRB5, INPP5D, MEF2C, NME8, PTK2B, SORL1, ZCWPW1, SlC24A4, CLU, PICALM, CR1, BIN1, MS4A, ABCA7, EPHA1, and CD2AP [28]. Oxidative stress and most common epigenetic modifications such as DNA methylation and histone post-translational modifications play critical roles in this disease [29,30,31]. Furthermore, except for genetic and epigenetic factors, some factors are believed to be associated with AD, such as age, race, hypertension, and diabetes. It is still unknown whether the appeared epigenetic changes directly lead to an effect of AD or not. One thing we can tell is that it is an extremely complicated disease, with numerous elements and factors involved in its progression and development. In order to investigate AD, there are lots of studies building networks based on omics data including genome, transcriptome, and proteome. One study combined gene expression profiles and genotyping data to construct molecular networks. Then, they applied network-based approach to rank-order these modules and identify key causal regulators by Bayesian inference [32]. Integrating human gene expression data and protein-protein interaction (PPI) data, Liu et al. tried to identify differentially activated pathways for six brain regions in AD, respectively, based on the proposed score scheme [33]. In addition, Li et al. discovered differentially expressed genes and built PPI networks to predict signaling pathways involved in AD by performing a statistical approach in meta-analysis [34]. However, there are few studies discussing AD from identifying essential biomarkers based on the molecular mechanisms to suggest potential multiple-molecule drugs in terms of engineering viewpoints.

**Figure 1:**
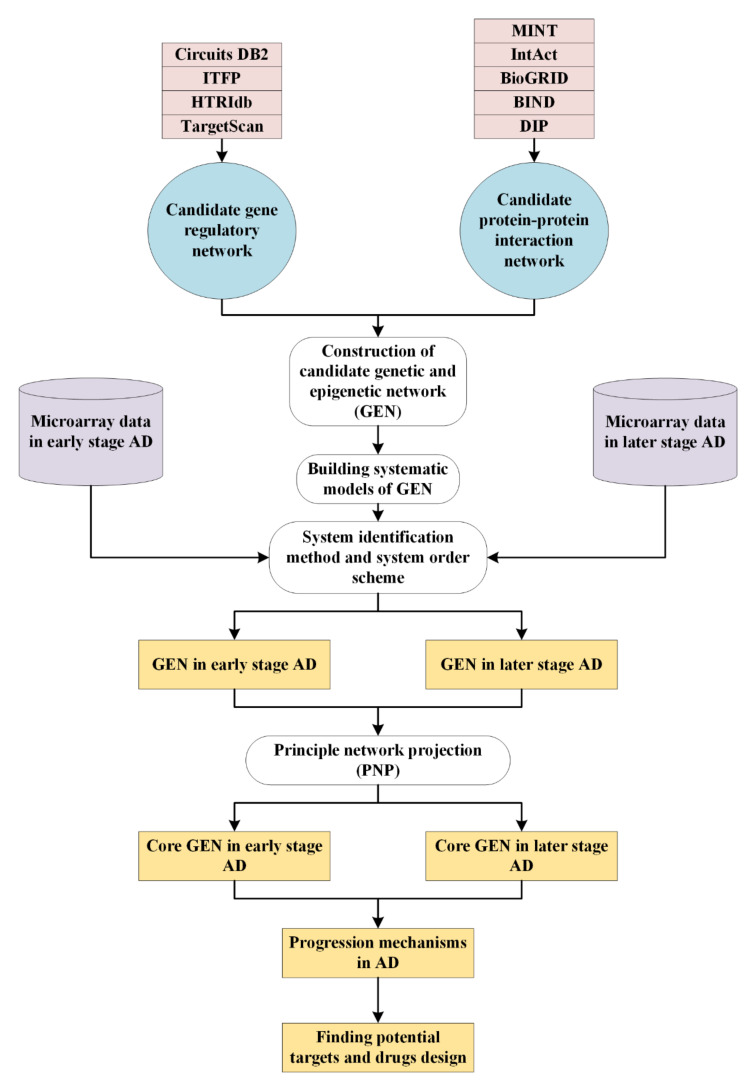
The research flowchart to investigate pathogenetic mechanisms of Alzheimer’s disease by systems biology approaches for drug discovery. The candidate genome-wide genetic and epigenetic network (GEN), which is composed of protein-protein interaction network (PPIN) and gene regulatory network (GRN), is built by mining big databases. The microarray data in early and later stage AD are shown in purple blocks. The rounded rectangular blocks indicate the schemes and methods utilized to construct the real and core GENs for early and later stage AD, respectively. The yellow blocks show the identified real GENs, core GENs, progression mechanisms, and the potential drug targets for the early and later stage AD.

Here, we provide systems biology approaches to investigate the pathogenic mechanisms of Alzheimer’s disease. The research flowchart is in Figure 1. Since the pathologic features are most limited to the medial temporal lobe structures in early stages (I-III); the neocortex is heavily affected in later stages (IV–VI) [2], we decided to separate AD samples into two groups: early stages (I–III) and later stages (IV-VI) in this study. Firstly, we built a candidate genome-wide genetic and epigenetic network (GEN) containing protein-protein network (PPI) and gene regulation network (GRN) by big databases mining. It is represented by a binary matrix. Assisted with microarray data in two stages of AD, we applied system identification via solving matrix inequality, a constrained linear least-squares parameter estimation problem, and performed system order detection scheme by evaluating Akaike information criterion (AIC) to prune false positives for obtaining real GENs. However, it is still too complicated to analyze real GENs. Therefore, we used principal network projection (PNP) method to extract core GENs from real GENs based on the projection values. Then, we could find core pathways by the annotation of KEGG pathways to investigate pathogenetic mechanisms of AD. Lastly, we identified several essential biomarkers and proposed two multiple-molecule drugs, aiming to interrupt disease progression or cure cognitive impairments for early and later stage AD, respectively.

## 2. Results

**Figure 2:**
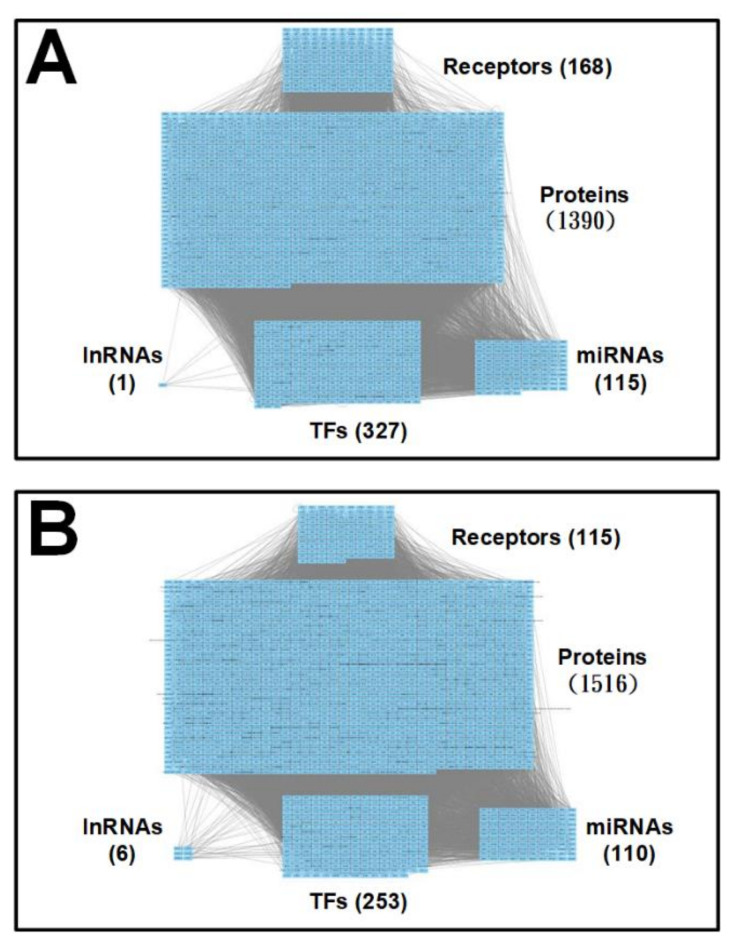
The identified genome-wide core GEN for (**A**) early-stage AD and (**B**) later stage AD. The core GENs are extracted from the real GENs (Figure S1) by the proposed principal network projection (PNP) method. The grey lines represent the interactions between groups, including receptors, proteins, miRNAs, TFs, and lncRNAs.

After building the candidate GEN by big database mining, system identification method and system order detection scheme were conducted to prune false-positive interactions in the candidate GEN to obtain real GENs with the help of microarray data of postmortem brain samples in early and later stage AD as shown in Figure S1. The identified real GENs of early and later stage AD are shown in Figure S1. We classify all of the nodes in GENs into five main groups: receptors, proteins, transcription factors (TFs), miRNAs and long noncoding RNAs (lncRNAs). The numbers of these identified nodes are also shown in Figure S1. Since the identified real GENs in both stages of AD were extremely complex, it was very difficult to investigate and discuss the precise pathogenic mechanism of AD directly. Therefore, we performed the principal network projection (PNP) method on the real GENs to extract core GENs. By utilizing the PNP method, we ranked all of the nodes from high projection value to low projection value. The nodes with top 2000 projection values were selected to be core nodes. Moreover, the core GENs consisting of these core nodes is shown in Figure 2. In addition, interactions and regulations of these core nodes in core GENs play important roles in the pathogenesis of AD. We further ran enrichment analysis by the Database for Annotation, Visualization and Integrated Discovery (DAVID) based on these core nodes of early and later stage AD to provide insights into cellular functions involved in the pathogenic mechanisms of AD. In the core GEN of early-stage AD, the enriched pathways are GABAergic synapse, Neurotrophin signaling pathway, Toll-like receptor signaling pathway, MAPK signaling pathway, and Wnt signaling pathway (Table S1). In the core GEN of later-stage AD, the enriched pathways are Jak-STAT signaling pathway, Apoptosis, Neurotrophin signaling pathway, TNF signaling pathway, and MAPK signaling pathway (Table S2). More details are given in the following sections. Furthermore, the aforementioned systems biology approaches, including system identification, systems order detection scheme, and PNP method is discussed in the Methods section.

### 2.1. Core Pathways in Early Stage AD

**Figure 3:**
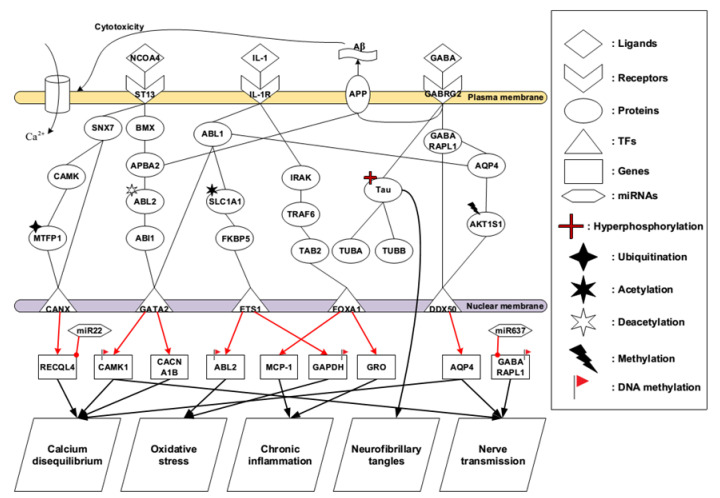
The core pathways in the early-stage AD. The core members were extracted from the core GENs in Figure 2A, showing the molecular mechanism to regulate the early events in AD. Calcium disequilibrium, oxidative stress, chronic inflammation, hyperphosphorylated tau protein, and nerve transmission are shown to onset in the very beginning of AD. The black lines indicate the protein-protein interaction; the red lines with arrowhead denote the regulation from upstream to downstream; the final cellular functions are mediated by target genes. Notations of genetic and epigenetic regulations are listed at the right rectangle.

The early-stage AD brain still remains normal physiological functions such as communication and neurotransmission. Several early events of AD occur simultaneously. The identified core pathways for the early-stage AD are shown in Figure 3. The ligand, NCOA4, binds to the receptor ST13 triggering two core pathways.

NCOA4, also named as ARA70, was described at first being a composition of RetFused Gene expressed in an experimental cohort of papillary thyroid carcinomas [35]. NCOA4 could be a coactivator response to various nuclear receptors [36], but we cannot exclude the possibilities that this coactivator may be a ligand for receptors on plasma membrane and involved in various signal transduction pathways to stimulate essential cellular function for cell survival. When NCOA4 forms a bond with ST13, it reveals the ability to participate in the constitution of glucocorticoid receptor and join the assistance of multiple molecular chaperones. Meanwhile, the downstream signaling is triggered by SNX7 and BMX for further cellular responses as shown in the identified core pathways in Figure 3. Looking to the first core pathway from the receptor ST13, the signaling is transmitted by SNX7, CAMK, and MTFP1 to the TF CANX. SNX7 family includes a phox region, which can be a region binding to phosphoinositide, and has intracellular trafficking ability [37]. CAMK protein kinases have appeared in various tissues and have been regarded as a member of a calmodulin-dependent protein kinase cascade. The alterations of CAMK signaling in AD have been elucidated, and calcium level in cytoplasm was elevated after Aβ being treated in the cultured neurons [38]. Combined with the activation of CAMK and the ubiquitination of MTFP1, the cascade activates the downstream TF CANX to regulate RECQL4, involving in calcium disequilibrium.

Looking into the second core pathway from the receptor ST13, the signaling istransmitted to the downstream TF GATA2 by BMX, APBA2, ABL2, and ABI1 as shown in Figure 3. Belonging to the Tec kinase family, BMX represents a non-receptor tyrosine kinase. This protein includes a domain with PH-like composition, which binds to phosphatidylinositol 3,4,5-triphosphate (PIP3), to activate membrane targeting and form a bond with SH2 domain, resulting in signal transduction by tyrosine-phosphorylated proteins. Implication of this protein has shown to have something to do with several signal transduction pathways, such as the Stat pathway, and tumorigenicity as well as differentiation, which are regulated by target enzyme in several types of cancer cells [39,40].

According to the identified core pathway in Figure 3, APBA2 can receive the signal coming from the BMX to trigger downstream signal transduction. It is noted that APBA2 can also interact with the APP by its neuronal adapter protein domain. One study indicated that original function of APBA2 is to stabilize APP and prevent formation of proteolytic products of APP including Aβ peptide [41]. Moreover, one of the most important pathological features in AD is plaque formation, while Aβ peptide is believed to be involved in biosynthesis of plaque. APP owns a transmembrane domain and appears in various organs, but predominantly concentrates in the brain. Another study has shown that proteolytic product of APP in normal conditions was cleaved by α-secretase followed by γ-secretase to generate non-amyloidgenic fragments [42]. Despite environmental factors, the proteolytic product of APP is changed, and β-secretase replaces α-secretase, and the residues is cleaved by γ-secretase to produce two main Aβ peptides, Aβ_40_ and Aβ_42_ [43]. In normal brain tissues, the major product of Aβ is Aβ_40_, while patients suffering from AD have a high ratio of Aβ_42_/Aβ_40_. Usually, Aβ_40_ can act as a nutritional factor and is non-toxic to the cells. By contrast, Aβ_42_ reveals more toxicity and more easily becomes aggregated than Aβ_40_. At higher concentrations of Aβ_42_, it irreversibly becomes aggregated and forms the insoluble amyloid plaque. During a person’s lifetime, numerous APP proteins are cleaved into Aβ in the intercellular space and the quaint balance between formation and reduction is mutually affected. Once the balance between production and clearance of these Aβ peptides is broken, aggregations cause plaque formation, resulting in the initiation of AD. This is referred to as the amyloid hypothesis [44].

The changed microenvironment such as ion concentration fluctuations may probably contribute to deactivating the property of APBA2 so that APP reveals the instability, producing a large amount of Aβ. Based on the core pathway analyses in Figure 3, we find that the deacetylation of ABL2 could be one of the turning points leading to the progression of AD. The deacetylated ABL2 could interact with ABI1 and then mediate the TF GATA2 to stimulate downstream cellular functions, resulting in calcium disequilibrium. Another identified important protein is AQP4, which is involved in ion concentrations, homeostasis and water molecules balance [45]. This protein is not only highly expressed in the brain, but also plays a critical role in brain water homeostasis. Our results speculate AQP4 and the methylation of AKT1S1 would contribute to maintaining cell communication and nerve transmission in brain tissues. Although the functions of APP are not very clear, our results suggest that GABRG2 may have some unknown relationships with APP. Under normal physiological conditions, γ-Aminobutyric acid (GABA) binds to its receptor GABRG2, triggering downstream signal transduction pathways to maintain communications between neurons and connected cells.

Meanwhile, it is known that the overproduction of Aβ leads to a neurotoxic effect on neurons [46]. It results in synaptic dysfunction and eventually leads to neuron loss. Aβ could interrupt the calcium channels in the cell membrane, so the equilibrium of calcium concentration between extracellular space and cytoplasm would undoubtedly be broken [47]. Thus, the influx of calcium is heavily increased, resulting in intracellular calcium overload. It will reduce the ability of mitochondria to cycle or buffer calcium, leading to cell toxicity and eventual cell death. The broken Ca^2+^ homeostasis may probably create a variety of adverse effects such as the production of free radicals and lipid peroxidation [48,49]. It has been verified that oligomeric types of Aβ_42_ create calcium-mediated toxicity in cultured cells. Degeneration of neurites appears in neurons related to Aβ depositions in mice with APP mutation, indicating the affection of calcium mediated Aβ neurotoxicity in vivo. Interestingly, a different mechanism showing Aβ may probably induce Ca^2+^ influx by inserting Aβ itself into the cell membrane and comprising pores similar to Ca^2+^ channels. Oligomeric Aβ reveals neurotoxicity in the brain. It has portions of functional and structural homology with the cytotoxic lymphocyte protein perforin and pore-forming bacterial toxins [50]. As the years go by, these related events of Aβ will break cell integrity and enhance neurotoxicity.

One of the most important pathological hallmarks is intracellular NFTs caused by hyperphosphorylated tau protein. The most widely known relationships are associated with disrupted tau and dysfunctional microtubules, which are referred to as the tau and tangle hypothesis [51]. In Figure 3, our speculated results showed that GABRG2 had some unknown interactions with tau protein. However, tau protein is neither regulated by upstream factors nor to mediate downstream signal transduction. The function of tau protein modulates the stability of axonal microtubules. There are two ways for tau to control microtubule stability: phosphorylation and changed structure. Tau proteins interact with tubulin to facilitate tubulin formation into microtubules and maintain the stability of microtubules [52]. In the brains of those with AD, the normal function of tau is gradually decreased so that tangles progressively replace the microtubules. Meanwhile, we also found that Tau has been adjusted by hyperphosphorylation in the identified core pathway. The hyperphosphorylated tau protein has low affinity binding to microtubules in this process. Elevated phosphorylation triggers tangle formation, and the aggregation of tau is shown to be isolated to the limbic system at an early stage. Mediated by local kinases and phosphatases, a balance has been shown between tau microtubule binding and process of withdrawing. This situation suggests that there is an uninterrupted change between a lowly phosphorylated state and a highly phosphorylated state.

One study has indicated that there was a relatively high concentration of IL-1 appearing in the cell lesions induced by Aβ cytotoxicity [53]. Notably, it is very unusual that such a high concentration of proinflammatory cytokine appeared in the brain. As a matter of fact, the microglia and astrocytes which are surrounded by Aβ plaques secrete inflammatory mediators IL-1, inducing innate immunity response in the lesions of injured cells. According to the identified core pathway in Figure 3, IL-1 directly binds to receptor IL-1R, triggering downstream early events in AD brain. Comparing with several lines of evidence that oxidative stress is produced due to ion oxidation, protein oxidation, DNA/RNA oxidation, and lipid peroxidation, our results show that signal transduction induced by IL-1R plays a pivotal role in regulating cellular function in this circumstance. Furthermore, ABL1, the previous function of this protein is a protein tyrosine kinase, which has various cellular responses, including cell division, adhesion, differentiation, and response to stress. In Figure 3, combined with the acetylation of SLC1A1 and the activation of FKBP5, ABL1 transmits the signal going through them to the downstream, which contributes to promoting the influence of oxidative stress. It probably would be one of the turning points leading to the progression of AD and causing far-reaching impact on the brain. In our results shown in Figure 3, miR-22 could regulate *RECQL4* gene, repressing AD early event, calcium disequilibrium. Interestingly, miR-22 has been identified to be a possibly neuroprotective miRNA due to its potential mediation of several targets involved in neurodegenerative disorders, such as Huntington’s disease [54].

### 2.2. Core Pathways in Later Stage AD

**Figure 4:**
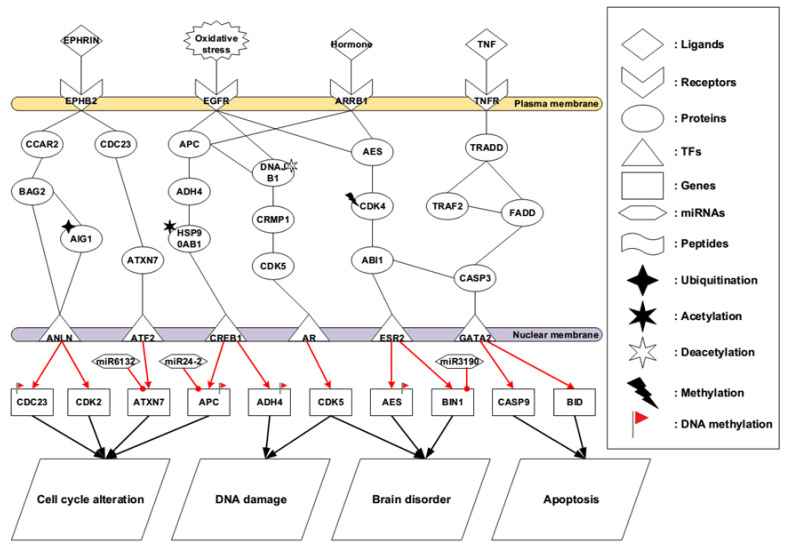
The core pathways in later stage AD. The core members were extracted from the core GENs in Figure 2B, showing the molecular mechanism to regulate the later events in AD. With the deterioration of the disease, the later stage AD brain is heavily influenced by multiple impacts including cell cycle alteration, DNA damage, brain disorder, and apoptosis. The black lines indicate the protein–protein interaction; the red lines with arrowhead denote the regulation from upstream to downstream; the final cellular functions are mediated by target genes. Notations of genetic and epigenetic regulations are listed at the right rectangle.

Compared to early-stage AD, later stage AD brains lose numerous pivotal biological functions such as communication, neurotransmission, energy metabolism, material transportation, proteins folding, and signal transduction due to extensively neuronal cell death in brain tissues. Therefore, the symptoms in later stage AD are usually very serious. Patients with severe AD have difficulties carrying out daily living activities, including driving, cooking, shopping, and taking care by themselves. As a result of long periods in a bed-ridden condition, pressure sores start to appear in multiple regions of the body with tissue necrosis. The identified core pathways for later stage AD are shown in Figure 4.

In later stage AD shown in Figure 4, EPHB2, a component of receptor tyrosine kinase transmembrane glycoproteins, belongs to the Eph receptor family. These receptors consist of an intracellular kinase domain, a transmembrane region, and an N-terminal glycosylated ligand-binding domain. When EPHB2 forms a bond with its ligand EPHRIN, a remarkable feature of Eph-ephrin signaling is that both ligands and receptors are capable of stimulating a signal transduction, leading to bidirectional signaling. EPHB2 is a member of a subset of the Eph receptors called EphB, giving two directions of regulation to mediate downstream regulators called CCAR2 and CDC23, as shown in the identified core pathways. Once CCAR2 is activated by the receptor EPHB2, it triggers the activation of BAG2 and then the ubiquitination of AIG1 provides the minor change in the microenvironment, thus enhancing the ability of TF ANLN. Evidence shows that AIG1 is an atypical hydrolytic enzyme that depends on the conserved threonine and histidine residues for catalysis [55]. Another identified core pathway is started by CDC23, which has shared significant resemblance with Saccharomyces cerevisiae Cdc23, a necessary element for passing through the G2/M transition in the cell cycle progression. This protein represents a synergistic reaction with anaphase-promoting complex, thus having the composition of eight protein subunits highly conserved in eukaryotic cells [56]. Therefore, both pathways are responsible for mediating cell cycle alteration via the influencing *CDK2* gene.

In Figure 4, the accumulative oxidative stress gives an impact on EGFR, causing downstream signal transduction. APC is responsible for catalyzing the constitution of cyclin B-ubiquitin conjugate that contributes to the ubiquitin-mediated proteolysis of B-type cyclins. The autosomal dominant cerebellar ataxias (ADCAs) have a heterogeneous characteristic of neurodegenerative diseases symbolized by degenerative development of the spinal cord, brain stem, and cerebellum. Heat shock protein HSP90AB1 is participated in signal transduction, protein folding and degradation and morphological evolution [57]. However, our results show that acetylation of HSP90AB1 may facilitate DNA double break causing by oxidative stress. Meanwhile, the deacetylation of DNAJB1 alters the role of the protein and regulates the downstream proteins CRMP1 and CDK5. CDK5 reveals a sequence resemblance similar to the other cell cycle kinases such as CDK2 and CDK1. Practically, CDK5 does not have a functionality involved in cell cycle to regulate or influence the activity of downstream mediators. On the contrary, it plays several roles in plastic synapse, cytoskeletal polypeptides phosphorylation, alive neurons, and developmental neurons. In our results, the deregulated activity of CDK5 has been involved in promoting AD [30], resulting in DNA damage so that DNA repair system is hard to recover to the original DNA sequence.

Due to numerous cell death in later stage AD brain tissues, various functional neurons lose their function, contributing to inducing vital hormones chaos. In Figure 4, these chaotic hormones influence receptor ARRB1, activating protein AES. Our speculated results suggest that CDK4 has altered basal level. Therefore, methylation of CDK4 may play a key role in this core signal transduction pathway. In this process, cellular communication may probably be interrupted, and nerve transmission could be broken. In the terminal of this identified core pathway shown in Figure 4, ABI1 interacts with ESR2 to regulate *AES* and *BIN1* genes, further promoting brain disorder. Evidence has shown that ABI1 is a key regulator of cytoskeletal reorganization during synaptic maturation and cellular migration [58]. 

Ligands of TNF family have shown the ability to induce the process of apoptosis in the central nervous system. It makes the brain vulnerable, leading to neurodegenerative disorders [59,60]. In the last core pathway in Figure 4, once ligand TNF binds to the receptor TNFR, TNFRSF1A associated via death domain (TRADD) regulates the signaling transduction of programmed cell death and the activation of NF-kappaB. On this identified core pathway, TRADD forms a bond with adaptor protein TRAF2, reducing the summoning of inhibitor-of-apoptosis proteins (IAPs) and downregulating TRAF2 to stimulate apoptosis. Meanwhile, TRAF2 would interact with FADD shown in Figure 4. One study has mentioned that TRADD had interaction with adaptor protein FADD/MORT1, involving in cell death pathway which is induced by Fas [61]. The activation of caspase-9 results from TNF mediated cell death due to the mitochondrial release of cytochrome-c. Moreover, according to the core pathways in the later stage AD (Figure 4), we found miR-24-2 could inhibit *APC* gene thus recovering cell cycle back to the normal condition. One study has indicated that miR-24-2 significantly deregulated in human glioblastoma tissues and glioblastoma cell lines [62].

### 2.3. Summary of Core Pathways in AD

**Figure 5:**
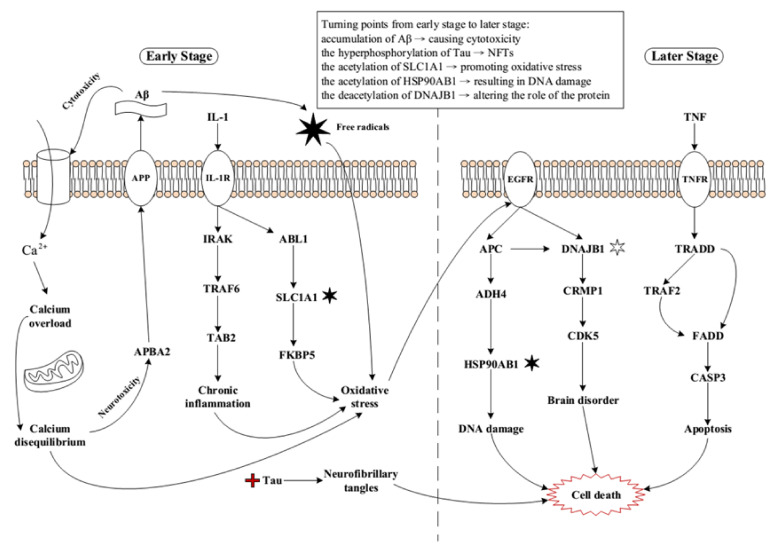
The overview of pathogenic mechanism of AD from early stage to later stage. The left part represents the pathogenic mechanism in early-stage AD, and right part indicates the pathogenic mechanism in later stage AD. The arrow lines denote the protein-protein interactions or regulations from the upstream to downstream. Notations of genetic and epigenetic regulations are the same as Figure 3 and Figure 4.

By applying systems biology approaches, we identified core pathways to investigate the pathogenic mechanisms of AD progression and development. Our findings have been summarized in Figure 5. One of the most important pathological features in AD is plaque formation, while Aβ peptide is believed to be involved in the biosynthesis of plaque. Meanwhile, the production and clearance of Aβ are retained in a delicate equilibrium. Probably, due to the minor changes of microenvironment, the delicate equilibrium of Aβ is broken irreversibly. Monomeric Aβ starts to aggregate, which goes through oligomeric units and finally forms insoluble Aβ plaque at higher concentrations. The insoluble Aβ plaque reveals a cytotoxic effect on neurons and interrupts the calcium channels on the cell membrane. Another mechanism shows that Aβ inserts itself into the cell membrane forming pores similar to Ca^2+^ channels. These actions lead to an increased Ca^2+^ influx from extracellular into intracellular, resulting in Ca^2+^ overload in the cytoplasm. Such high concentrations of Ca^2+^ will reduce the ability of mitochondria to cycle or buffer calcium, causing cell toxicity and, thus, changing the microenvironment in the cytoplasm. Therefore, it is referred to calcium disequilibrium, resulting in a huge impact on microenvironment of cytoplasm.

The original function of APBA2 is to stabilize APP and prevent the formation of proteolytic products of APP including Aβ peptide. Since the equilibrium between Ca^2+^ and other ions has been interrupted, the protein APBA2 no longer has the ability to stabilize APP, thus producing a large amount of Aβ. That is, a vicious circle is forming and the situations in early-stage AD brain are seen to be out of control. Meanwhile the microglia and astrocytes which are surrounded by Aβ plaques secrete inflammatory mediators IL-1, inducing innate immunity response in the lesions of injured cells. Once IL-1 binds to the interleukin-1 receptor (IL1R), IL1R stimulates downstream signal complex, which is composed of TRAF6 and TAB2. This signal transduction pathway induced by IL-1 and IL1R further regulates the gene expression of MCP-1 and GRO [63], resulting in a stronger inflammatory response in the early-stage AD brain.

ABL1 is a protein tyrosine kinase associated with various cellular responses, including cell division, adhesion, differentiation, and response to stress [64]. Stimulated by IL-1R, ABL1 triggers downstream regulation. Increased oxidative stress is induced by the lack of SLCIA1 in nullizygous mice [65]. Our results indicate that the acetylation of SLC1A1 could be one of the turning points in early-stage AD to promote oxidative stress. Furthermore, two cascades associated with oxidative imbalance have been built up. Chronic inflammation induced by IL-1 proinflammatory cytokine and calcium disequilibrium with dysfunctional mitochondria lead to accumulating oxidative stress. Another important cascade is that Aβ produces ROS and free radicals, which are inevitable metabolic products and have been seen to be a double-edged axe in our metabolic system. At the same time, it generates high oxygen consumption, resulting in oxidative stress undoubtedly.

Epidermal growth factor receptor (EGFR) represents one type of transmembrane glycoprotein, which is a subset of the protein kinase superfamily. It is involved in various biological functions and is highly influenced by oxidative stress. When this protein forms a bond with a ligand, it triggers receptor dimerization and tyrosine autophosphorylation, thus leading to downstream regulations. APC acts as a protein of tumor inhibition, which encodes an antagonist responding to Wnt signaling pathway. Several biological processes including cell adhesion, transcriptional activation, migration, and apoptosis are found to be associated with this protein. Evidence shows that defects in this protein cause familial adenomatous polyposis (FAP) [66], thus giving the essential ability in related cascade. Members of ADH4 family catalyze a wide variety of substrates including retinol, ethanol, other aliphatic alcohols, hydroxysteroids, and lipid peroxidation products, evidently linking to relevant impact of oxidative imbalance. Subsequently, the activated ADH4 leads to the acetylation of HSP90AB1, resulting in DNA double break and DNA damage. They are hard to be recovered via DNA repair system, giving rise to a permanent damage in the process.

Meanwhile, CRMP1 is activated by the deacetylation of DNAJB1 to mediate the activity of CDK5. Impaired CDK5 loses its original functions such as plastic synapse, cytoskeletal polypeptides phosphorylation, alive neurons, and developmental neurons, directly contributing to multiple brain disorders and interrupted communication between pivotal organelles. Studies indicate that a large number of neuronal losses occur in later stage AD due to apoptosis, which is triggered by TNF signaling pathway [67,68,69].

Finally, three later events in AD including DNA damage, brain disorder, and apoptosis are responsible for neuronal cell death and irreversibly deteriorate the progression of disease. Here, we summarize several critical turning points from early stage to later stage: accumulation of Aβ → causing cytotoxicity, the hyperphosphorylation of tau → NFTs, the acetylation of SLC1A1 → promoting oxidative stress, the acetylation of HSP90AB1 → resulting in DNA damage, and the deacetylation of DNAJB1 → altering the role of the protein.

### 2.4. Multiple-Molecule Drug Design for the Identified Biomarkers in Early and Later Stage AD

According to core pathways in early-stage AD, we selected the overlap nodes with Connectivity Map (CMap) [70] to be the identified biomarkers as drug targets. CMap is a large-scale perturbation database. It contains transcriptomic profiles in dozens of cultivated cell lines treated with thousands of chemical compounds. The goal here is to find drugs, which could target identified biomarkers as many as possible and reverse the abnormal gene expression. For instance, a positive coefficient denotes the degree of similarity, and a negative coefficient emphasizes an opposite similarity between a query signature and a reference profile derived from a chemical perturbation [71]. Moreover, we took the top three small molecules, which are satisfied with our expectations (targets to be inhibited or targets to be enhanced), to be the potential drug candidates.

In early-stage AD, ST13, IL-1R, CAMK1, SLC1A1, AQP4, TAB2, GABRG2, and APBA2 were identified to be the significant biomarkers. ST13 has revealed the ability to participate in the assembly process of glucocorticoid receptor and has a ligand binding domain. IL-1R is responsible for neuroinflammation induced by proinflammatory cytokine IL-1, which is secreted from microgila to lesions of cell. CAMK1 regulates transcription activator activity, cell cycle, hormone production, cell differentiation, actin filament organization and neurite outgrowth upon calcium influx, thus being very critical because of calcium equilibrium. Based on our results, acetylated SLC1A1 plays a pivotal role as a mediator in IL-1 induced oxidative stress pathway. Moreover, TAB2, forming a kinase complex with TRAF6, serves as an adaptor protein. AQP4 is involved in ion concentration homeostasis and water molecule balance. In order to control the water pressure in the brain, the water flow from extracellular space into the cytoplasm should be administered to avoid brain edema. Therefore, according to their biological functions or their roles in the identified core pathways, the activities of these six proteins should be repressed. On the other hand, GABRG2 is important for cell communication and neurotransmission. Contributing to stabilizing the activity of APP, APBA2 could prevent the overproduction of Aβ. Thus, the activities of GABRG2 and APBA2 should be activated. By querying CMap, small molecules that achieve the remedy screening criterion would be selected to be the potential candidate drugs. We proposed a potential multiple-molecule drug including duloxetine, sulindac, and avagacestat to treat early-stage AD. Moreover, duloxetine is evaluated for tolerability and efficacy in the treatment of major depressive disorder and associated physical symptoms [72]. Sulindac is one of the nonsteroidal anti-inflammatory drugs, which has the activity of selective *γ*-secretase modulators to inhibit Aβ toxicity forming [73]. Avagacestat shows safety and tolerability in the treatment of mild to moderate AD and has been conducted in phase 2 clinical trials [74]. The proposed multiple-molecule drug for the early-stage AD is given in Table 1.

In later stage AD, we identified EPHB2, EGFR, ARRB1, TNFR, FADD, APC, CDK5, and AIG1 to be the significant biomarkers. EPHB2 is a component of the Eph receptor family of receptor tyrosine kinase transmembrane glycoproteins, which is activated by a ligand EPHRIN. EGFR acts as a membrane protein binding to epidermal growth factor, and is highly influenced by oxidative stress, thus triggering downstream signal transduction. ARRB1 acts as an activator to stimulate beta-adrenergic receptor kinase (BARK). TNFR plays an important role in the apoptosis pathway. Meanwhile, FADD is a critical mediator to regulate downstream cascade. Therefore, according to their biological functions or their roles in the identified core pathways, the activities of these five proteins should be repressed. On the other hand, the ubiquitination of AIG1 and CDK5 has a significant basal level because of the role of the proteins. The APC protein is a negative regulator that controls beta-catenin concentrations and interacts with E-cadherin. Thus, the activities of AIG1, APC, and CDK5 should be activated. By querying CMap, small molecules satisfying the remedy screening criterion would be selected as the potential candidate drugs. We proposed a potential multiple-molecule drug including trazodone, tideglusib, and epothilone D to treat later stage AD. Furthermore, trazodone is a triazolopyridine antidepressant, which has been the second most commonly prescribed agent in the treatment of insomnia because of its sedating qualities [75]. Tideglusib, an inhibitor of glycogen synthase kinase-3, shows the safety and tolerability in the treatment of AD and produces positive trends in the mini-mental status examination (MMSE) in small samples [76]. Epothilone D has been regarded as the brain-penetrant microtubule-stabilizing agent. It could increase axonal microtubule density and reduce axonal dystrophy in the aged mice, improving fast axonal transport and cognitive performance [77]. The proposed multiple-molecule drug for the later stage AD is shown in Table 2.

Advanced AD drug candidates are few compared with those for other diseases in the development progress [78]. Discovering a very large number of biological targets is in need nowadays. Among the suggested drug candidates in Table 1 and Table 2, it is noted that Duloxetine, Sulindac, and Trazodone have been approved by the FDA. Although they are not approved for the efficacy of treating AD, they probably would help with drug repurposing, which is a strategy for identifying new uses to old drugs. Moreover, some clinical trials indicated that Avagacestat and Tideglusib had good tolerability but no clinical benefits under certain doses in AD patients [79,80]. A phase I clinical trial of low dose Epothilone D for the treatment of patients with mild AD was discontinued [81]. However, we still believe that further dosage, timing, and their synergistic effect are worth studying for these proposed multiple-molecule drug candidates. For instance, one study has demonstrated that the average transport speed of mitochondria would be affected by the different concentrations of Epothilone D [82]. The systems biology approach-based analyses in this study might provide a new perspective of finding multitarget-directed drug candidates for the therapeutics of AD.

## 3. Discussion

### 3.1. Identified Pathogenetic Mechanisms in Early Stage AD

NFTs and Aβ peptides are two important pathological features in AD brain. A staging method has been proposed in 1991 based on NFTs appearing in several brain regions, so it is impressed that the AD stages have been classified into three classical stages: transentorhinal stage, limbic stage, and isocortical stage. However, one review report indicates that the pathologic hallmarks are most limited to the medial temporal lobe structures in early stages (I–III); the neocortex is heavily affected in later stages (IV–VI) [2]. Therefore, in this study we divided all post-mortem brain samples into two groups: early stage (I-III) and later stage (IV–VI). Interestingly, in spite of largely knowing about the protein components of both Aβ peptides and NFTs, the comprehensively detailed formation remains elusive. Two famous hypotheses have been proposed to give an explanation about the pathogenic progression mechanisms of AD: amyloid hypothesis [44] and tau and tangle hypothesis [51]. Advocates of these two hypotheses have debated for a long time. According to Braak’s staging method, more and more areas of the brain are affected over time. However, whether the hyperphosphorylated tau that propagates from other affected neurons is merely endogenous or exogenous is still a matter of debate. Evidence has shown that one uncommon type of soluble phosphorylated tau was involved in propagation and taken by neurons in mice model [83]. Although Aβ hypothesis has been the predominate statement to explain the pathogenic progression mechanism of AD, advocates of the tau and tangle hypothesis still do their best to prove themselves. All of these findings will continuously drive experts to explore more significant outcomes for further studies.

Extensive literatures support a role for oxidative damage in the pathogenesis of AD. Before the onset of significant plaque pathology, it is believed that oxidative damage is an early event in AD. Elements receiving the most attention in AD have been ion oxidation, protein oxidation, DNA/RNA oxidation, and lipid peroxidation. In our results, acetylation of SLC1A1 may probably be the end product of protein oxidation (Figure 3). Moreover, studies show that Aβ peptide is capable of generating free radicals. It not only involves in cell chemical toxicity but also gives rise to oxidative damage on neurons, mediating neuron degeneration and death (Figure 5). Along with the massive oxygen consumption by the brain, reactive oxygen species (ROS) can possibly contribute to neuronal damage in aging and neurological disorders for AD. Not surprisingly, the subsequent oxidative stress and oxidative imbalance, regulating damage to molecules, are widely found in AD. ABL1, a protein tyrosine kinase, is involved in a variety of cellular processes, including cell division, adhesion, differentiation, and response to stress. Hence, this protein reveals high sensitivity to ROS (Figure 3). FKBP5 is a member of the immunophilin protein family, which plays roles in immunoregulation and basic cellular processes involving protein folding and trafficking. Interestingly, in our results, FKBP5 receives signals from the acetylation of SLC1A1, contributing to producing oxidative stress. Namely, the active members in immunity and oxidative imbalance have a close relationship with each other (Figure 3). CAMK1 operates in the calcium-triggered signaling cascade, thus calcium overload has multiple impacts on the calcium regulatory pathway. Studies have suggested that elevated CAMK1 would contribute to elevating intracellular calcium levels in early-stage AD brains [84,85]. Moreover, oxidative imbalance causing RNA/DNA breaks is greatly enhanced in AD brains [86,87]. Attacked by oxidative stress, DNA may have DNA double strand breaks, base modification, and DNA/protein crosslinking (Figure 4). Although DNA repair systems could be activated soon after the event occurs and the broken DNA may result in irreversible damage causing downstream adverse reactions.

Brain inflammation is a characteristic feature in AD. It is regarded as an early event since the ongoing deposition of Aβ starts to aggregate gradually. However, the classical hallmarks of inflammation including pain, heat, and swelling are not shown in the brain. Therefore, it is clear that we attribute this condition to chronic inflammation instead of acute inflammation (Figure 3). Three main cells are involved in the inflammatory process in AD: microglia, astrocytes, and neurons. Once these cells are activated, microglia and astrocytes generate several proinflammatory signal molecules, including cytokines (IL-1) (Figure 3), chemokines, growth factors (hormone) (Figure 4), cell adhesion molecules, and complement molecules. The role of phagocytic macrophages is very similar to the character performed by microglia in central nervous system, which acts as the first defensive wall against exogenous pathogens or other forms of brain tissue injury [88]. IRAK is one of two putative serine/threonine kinases that become associated with the interleukin-1 receptor (IL1R) upon stimulation. TRAF proteins mediate signal transduction from elements of the TNF receptor superfamily. This protein also has interactions with protein kinases such as IRAK that produce a connection with distinct signaling pathways. TAB2 forms a kinase complex with TRAF6, MAP3K7 and TAB1, and it thus serves as an adaptor that links MAP3K7 and TRAF6. Our results could be obviously proved by the properties of these three proteins shown in Figure 3. Cytokine production needs a key element called nuclear factor-kappaB (NFκB) to trigger the corresponding dependent pathway, meanwhile Aβ is able to act as a stimulator. When chemokines and cytokines are produced and proinflammatory gene expression is activated by Aβ binding to the microglial cell membrane, the following reactions of mitogen-activated protein kinase (MAPK) and extracellular signal-regulated kinase (ERK) pathways are activated [89]. In Tables S1 and S2, the enriched pathway analysis of core GEN in early and later stage AD reveals MAPK signaling pathway, which is verified by the aforementioned study. Furthermore, the level of cytokines dominates remarkable changes in AD cerebrospinal fluid and brain tissues [90,91]. Two agonist proteins, IL-1α and IL-1β, belong to the interleukin 1 (IL-1) family of cytokines. They could induce cell activation when specific membrane proteins are forming a bond with IL-1 (Figure 3). IL-1 is associated with the process of initiation and propagation in AD pathological changes. An overproduction of IL-1 has been found to trigger the inflammatory process. Once a vicious circle is produced by the overload of IL-1, it may lead to neuronal dysfunction and death.

### 3.2. Identified Pathogenetic Mechanisms in Later Stage AD

It is believed that various balances and checks regulate the process of cell cycle, so that when suitable factors are present in the environment, the normal cells in the brain can proliferate appropriately. The cell cycle classically has four phases: G_1_, S (DNA replication), G_2_ and M (mitosis). In normal brains, the primary neurons are seen to be in G_0_ and quiescent state. EPHB2 receptor family is composed of an N-terminal glycosylated ligand-binding domain, a transmembrane region, and an intracellular kinase domain. They bind ligands, ephrins, which are involved in diverse cellular processes, including motility, division, and differentiation (Figure 4). Probably, due to the ubiquitination of AIG1, the cell cycle seems to be out of control, resulting in cell cycle alteration. Our results show that ATXN7 could be self-regulation in this pathway, thus promoting cell cycle alteration. It has been proved that ATXN7 was a heterogeneous group of neurodegenerative disorders characterized by the progressive degeneration of cerebellum, brain stem and spinal cord. The major mediators of cell cycle progression, cyclin/cyclin-dependent kinase (CDK) complexes, activate the sequential expression of signal pathways. Cells could go out of cell cycle moving on and stop at G_0_ phase after the mitosis has been finished. Neurons, which are highly differentiated cells, belong to this case. Next, mitotic growth factors trigger cyclin D/CDK4,6 complex to be activated, controlling the cell cycle from G_0_ phase into G_1_ phase [92]. It is reported that the initiation of cell cycle processes may activate the mechanism of programmed cell death of defective neurons due to their developmental failure of trophic support. Additionally, one study has suggested that various stressors might evoke neuronal death mediated by cell cycle regulators [93] (Figure 4).

DNA damage is related to aging, which is a complicated process that several factors and elements are involved in. In human beings, DNA damage induced by oxidative imbalance is detected in the brain tissues during the process of aging. EGFR is a very sensitive receptor, and this transmembrane glycoprotein is a member of the protein kinase superfamily. EGFR would bind to epidermal growth factor. Moreover, a ligand interacting with the protein induces receptor dimerization and tyrosine autophosphorylation and leads to cell proliferation. Once this process is interrupted by oxidative stress, it can irreversibly cause DNA damage and DNA double strand break. ADH4 is an alcohol dehydrogenase, which is a member of the alcohol dehydrogenase family. When the protein conducts redox reaction, ROS may be generated, enhancing oxidative imbalance. The tight regulation of CDK5 is disrupted under many neurotoxic or stress conditions. Therefore, hyperactivity of CDK5 is involved in promoting cell death via a feedback loop mechanism by an upstream regulator as well as a downstream effector (Figure 5).

Apoptosis is a pivotal cellular function symbolizing a series of biochemical processes. Receiving definite stimuli from exogenous and endogenous mediators from a cell, the programmed cell death is provoked, causing the cell to proceed towards the end of activity. Although numerous works have been undertaken in order to understand the apoptotic mechanisms, an effective therapeutic strategy that may slow or stop this process still remains obscure. The programmed cell death is triggered by several regulators including Par-4, Bax, BCL-XS, caspases, p53, c-Jun N-terminal kinases, p38, p21, and mitogen-activated protein kinase (MAPK) [94,95]. As a matter of fact, apoptosis has a high relationship with MAPK signaling pathway.

## 4. Materials and Methods

### 4.1. Overview of Systems Biology Approaches to Investigate Pathogenetic Mechanisms of AD

To elucidate the overall systems biology approaches, we provide a general research flowchart for understanding how the proposed methods contribute to constructing core GENs and analysing core pathways with pathogenetic mechanisms (Figure 1). In this study, we could divide the overall systematic processes into several steps. At first, the candidate GEN including PPIN and GRN should be built by big databases mining. It is noted that there are lots of false-positive interactions and regulations in the candidate GEN caused by various experimental conditions and noises. Therefore, we have to prune these false positives for obtaining real GENs in early and later stage AD. Applying systems identification method, unknown parameters in system models can be estimated by solving constrained linear least-squares problem with the help of microarray data in early and later stage AD. Furthermore, we compute Akaike information criterion (AIC) to detect the real system order. In other words, insignificant interactions and regulations, which are out of the real system order would be pruned away. However, these real GENs of early and later stage AD are still too complex to specifically analyze the pathogenic mechanisms of AD. Here, we utilize PNP method to extract core GENs. Sorting projection value from greatest to least, key core pathways annotated in KEGG pathway style are picked out from core GENs. Consequently, based on the pathogenetic mechanisms analyses of AD and finding the overlap nodes with CMap, we identify essential biomarkers and propose two multiple-molecule drugs to interrupt disease progression and development in AD.

### 4.2. Datasets

For building the candidate GEN, we collect interactions and regulations data from the related database: DIP [96], BIND [97], BioGRID [98], IntAct [99], MINT [100], CircuitDB2 [101], TargetScan [102], ITFP [103], Transfac [104], HTRIdb [105]. The candidate GEN is a binary matrix. If there exists an interaction or a regulation, we will give one, vice versa we would give zero. The microarray data, which we use to perform systems identification and systems order detection scheme for obtaining real GENs of AD, could be downloaded from National Center for Biotechnology Information Search database (NCBI) website (https://www.ncbi.nlm.nih.gov/, accessed on 19 October 2021). The accession number is GSE84422. Based on the braak neurofibrillary tangle score derived in the GEO for each sample, we divide all AD samples into two groups: early stages (braak neurofibrillary score: I–III) and later stages (braak neurofibrillary score: IV–VI). Early and later groups have 117 and 263 postmortem brain samples, respectively. 125 human brains were accessed from the Mount Sinai/JJ Peters VA Medical Center Brain Bank (MSBB). There are 19 cortical regions in 125 individuals’ brain sample having variety of neuropathology and dementia of AD severely. Despite of enormous discrepancy among individuals with regard to pathological progression and development, the experimental groups that we set up are based on neuropathologic changes associated with Braak’s staging [7] rather than clinical symptoms.

### 4.3. Systems Modeling for the Candidate GEN

Before performing systems identification and system order detection scheme, we do system modeling for proteins, genes, miRNA, and lncRNAs. The protein-protein interactions (PPIs) in candidate PPIN, which is a sub-network of candidate GEN, could be described in the following Equation:

(1) $$p_{i}\left[n\right]=\overset{M_{i}}{\underset{\begin{array}{c} m=1\\ m\neq i \end{array}}{\sum }}a_{im}p_{i}\left[n\right]p_{m}\left[n\right]+\beta_{i,PPIN}+\varepsilon_{i,PPIN}\left[n\right],\;for\;i=1,\ldots ,I,\;n=1,\ldots ,N$$

where the expression levels of the ith protein and mth protein for the *n*th sample are denoted by $p_{i}\left[n\right]$ and $p_{m}\left[n\right]$, respectively. Besides, $a_{im}$ is the interaction ability between the ith protein and mth protein; *I* indicates the total number of proteins and *N* shows the number of patient samples; $\beta_{i,PPIN}$ stands for the basal level of the *i*th protein; $\varepsilon_{i,PPIN}\left[n\right]$ denotes the stochastic noise because of the modeling residue and fluctuation in the ith protein. Observing basal level change higher than a threshold, we infer that the *i*th protein is influenced by epigenetic modifications, such as acetylation, phosphorylation, and ubiquitination.

Similarly, gene regulation network (GRN), which is a sub-network in the candidate GEN, consists of genes and their regulatory TFs/proteins, lncRNAs and miRNAs. In order to describe the overall genetic regulatory network in the candidate GRN, we would undertake system modeling for them separately. In the following, the systematic gene regulatory model is given as below:

(2) $$g_{j}\left[n\right]=\overset{S_{j}}{\underset{s=1}{\sum }}b_{js}p_{s}\left[n\right]+\overset{T_{j}}{\underset{t=1}{\sum }}c_{jt}x_{t}\left[n\right]-\overset{U_{j}}{\underset{u=1}{\sum }}d_{ju}r_{u}\left[n\right]+\beta_{j,GRN}+\varepsilon_{j,GRN}\left[n\right],\;\mathrm{for}\;j=1,\ldots ,J,\;n=1,\ldots ,N,\;d_{ju}>0$$

where $g_{j}\left[n\right]$ is the expression level of the *j*th gene; $p_{s}\left[n\right]$, $x_{t}\left[n\right]$ and $r_{u}\left[n\right]$ represent the expression levels of the *s*th TF/protein, the *t*th lncRNA and the *u*th miRNA, respectively; $b_{js}$ denotes the transcription regulatory ability of the *s*th TF to the *j*th gene; $c_{jt}$ is the transcription regulatory ability from the *t*th lncRNA to the *j*th gene; $d_{ju}$ indicates the post-transcriptional regulatory ability of the *u*th miRNA to inhibit the *j*th gene; $S_{j}$ is he total number of TFs binding to the *j*th gene; $T_{j}$ denotes the total number of lncRNAs binding to the *j*th gene; $U_{j}$ stands for the total number of miRNAs inhibiting the *j*th gene; $\beta_{j,GRN}$ is the basal level of the *j*th gene; $\varepsilon_{j,GRN}\left[n\right]$ is regarded as stochastic noise coming from the modeling residue and fluctuation to the *j*th gene; J and N indicate the number of genes and patient samples, respectively. Moreover, the details of systems modeling for miRNAs and lncRNAs can be found in the Supplementary Materials.

### 4.4. Systems Identification to the Candidate GEN via Microarray Data of Early and Later Stage AD

With the help of microarray data, we used the systems identification method to estimate the interactive and regulatory parameters. The Equation (1) can be rewritten as below:

(3) $$p_{i}\left[n\right]=\left[\begin{array}{cc} p_{1}\left[n\right] & \begin{array}{ccc} p_{2}\left[n\right] & \cdots & \begin{array}{cc} p_{M_{i}}\left[n\right] & 1 \end{array} \end{array} \end{array}\right]\cdot \left[\begin{array}{c} \begin{array}{c} a_{i1}\\ a_{i2} \end{array}\\ \vdots \\ a_{iM_{i}}\\ \beta_{i,PPIN} \end{array}\right]+\varepsilon_{i,PPIN}\left[n\right]\;\equiv \phi_{i,PPIN}^{T}\left[n\right]\cdot \theta_{i,PPIN}+\varepsilon_{i,PPIN}\left[n\right],\;\mathrm{for}\;i=1,\ldots ,I$$

where $\phi_{i,PPIN}^{T}\left[n\right]$ denotes the regression vector which we could get from the corresponding microarray data. The vector $\theta_{i,PPIN}$ denotes the protein interaction parameters which have to be estimated. Moreover, there are N samples of AD microarray data. Therefore, the Equation (3) can be augmented in the following form:

(4) $$\left[\begin{array}{c} p_{i}\left[1\right]\\ \vdots \\ p_{i}\left[N\right] \end{array}\right]=\left[\begin{array}{c} \phi_{i,PPIN}^{T}\left[1\right]\\ \vdots \\ \phi_{i,PPIN}^{T}\left[N\right] \end{array}\right]\cdot \theta_{i,PPIN}+\left[\begin{array}{c} \varepsilon_{i,PPIN}\left[1\right]\\ \vdots \\ \varepsilon_{i,PPIN}\left[N\right] \end{array}\right],\;\mathrm{for}\;i=1,\ldots ,I$$

Which can be simplified by:

(5) $$P_{i}=\Phi_{i,PPIN}\cdot \theta_{i,PPIN}+{Ε}_{i,PPIN}$$

where $P_{i}=\left[\begin{array}{c} p_{i}\left[1\right]\\ \vdots \\ p_{i}\left[N\right] \end{array}\right],\;\Phi_{i,PPIN}=\left[\begin{array}{c} \phi_{i,PPIN}^{T}\left[1\right]\\ \vdots \\ \phi_{i,PPIN}^{T}\left[N\right] \end{array}\right],\;{Ε}_{i,PPIN}=\left[\begin{array}{c} \varepsilon_{i,PPIN}\left[1\right]\\ \vdots \\ \varepsilon_{i,PPIN}\left[N\right] \end{array}\right]$

To estimate the interactive parameters in the vector $\theta_{i,PPIN}$, we have to solve the linear least-squares estimation problem shown in the Equation (6).


(6) $${\hat{\theta }}_{i,PPIN}=\underset{\theta_{i,PPIN}}{\mathrm{argmin}}\frac{1}{2}\Phi_{i,PPIN}\cdot \theta_{i,PPIN}-P_{i}\overset{2}{\underset{2}{}}$$

The aforementioned estimation problem is solved by utilizing optimization toolbox function *lsqlin* in MATLAB.

In the same way, the gene regulatory Equation in (2) could be rewritten as follows:

(7) $$\begin{array}{ll} g_{j}\left[n\right] & =\left[p_{1}\left[n\right]\;\cdots \;p_{S_{j}}\left[n\right]\;x_{1}\left[n\right]\;\cdots \;\;x_{T_{j}}\left[n\right]\;\;r_{1}\left[n\right]\;\cdots \;r_{U_{j}}\left[n\right]\;\;1\right]\cdot \left[\begin{array}{c} b_{j1}\\ \vdots \\ \begin{array}{c} b_{jS_{j}}\\ c_{j1}\\ \begin{array}{c} \vdots \\ c_{jT_{j}}\\ \begin{array}{c} -d_{j1}\\ \vdots \\ \begin{array}{c} -d_{j_{U_{j}}}\\ \beta_{j,GRN} \end{array} \end{array} \end{array} \end{array} \end{array}\right]+\varepsilon_{j,\;GRN}\left[n\right]\\ & \equiv \phi_{j,GRN}^{T}\left[n\right]\cdot \theta_{j,GRN}+\varepsilon_{j,GRN}\left[n\right],\;\mathrm{for}\;j=1,\;\ldots ,\;J. \end{array}$$

where $\phi_{j,GRN}^{T}\left[n\right]$ indicates the regression vector, which could be obtained from the microarray data. $\theta_{j,GRN}$ is a parameter vector, which have to be estimated for the *j*th gene in the GRN. For the N samples in AD microarray data, we get

(8) $$\left[\begin{array}{c} g_{j}\left[1\right]\\ \vdots \\ g_{j}\left[N\right] \end{array}\right]=\left[\begin{array}{c} \phi_{j,GRN}^{T}\left[1\right]\\ \vdots \\ \phi_{j,GRN}^{T}\left[N\right] \end{array}\right]\cdot \theta_{j,GRN}+\left[\begin{array}{c} \varepsilon_{j,GRN}\left[1\right]\\ \vdots \\ \varepsilon_{j,GRN}\left[N\right] \end{array}\right],\;\mathrm{for}\;j=1,\ldots ,J$$

Which could be simply shown as:

(9) $$G_{j}=\Phi_{j,GRN}\cdot \theta_{j,GRN}+{Ε}_{j,GRN}$$

where $G_{j}=\left[\begin{array}{c} g_{j}\left[1\right]\\ \vdots \\ g_{j}\left[N\right] \end{array}\right],\;\Phi_{j,GRN}=\left[\begin{array}{c} \phi_{j,GRN}^{T}\left[1\right]\\ \vdots \\ \phi_{j,GRN}^{T}\left[N\right] \end{array}\right],\;{Ε}_{j,GRN}=\left[\begin{array}{c} \varepsilon_{j,GRN}\left[1\right]\\ \vdots \\ \varepsilon_{j,GRN}\left[N\right] \end{array}\right]$.

Hence, by solving the following constrained linear least-squares estimation problem in Equation (10), we could estimate the regulatory parameters in the vector $\theta_{j,GRN}$.

(10) $${\hat{\theta }}_{j,GRN}=\underset{\theta_{j,GRN}}{\mathrm{argmin}}\frac{1}{2}\|\Phi_{j,GRN}\cdot \theta_{j,GRN}-G_{j}\|\overset{2}{\underset{2}{}}$$

$$\mathrm{subject}\;\mathrm{to}\;\left[\begin{array}{ccc} \overset{S_{j}+T_{j}}{\overset{︷}{\begin{array}{ccc} \begin{array}{c} 0\\ \vdots \\ 0\\ \begin{array}{c} 0\\ 0 \end{array} \end{array} & \begin{array}{c} \cdots \\ \vdots \\ \cdots \\ \begin{array}{c} \cdots \\ \cdots \end{array} \end{array} & \begin{array}{c} 0\\ \vdots \\ 0\\ \begin{array}{c} 0\\ 0 \end{array} \end{array} \end{array}}} & \overset{U_{j}}{\overset{︷}{\begin{array}{cccc} \begin{array}{c} 1\\ \vdots \\ 0\\ \begin{array}{c} 0\\ 0 \end{array} \end{array} & \begin{array}{c} \cdots \\ \ddots \\ \cdots \\ \begin{array}{c} \cdots \\ \cdots \end{array} \end{array} & \begin{array}{c} 0\\ \vdots \\ 1\\ \begin{array}{c} 0\\ 0 \end{array} \end{array} & \begin{array}{c} 0\\ \vdots \\ 0\\ \begin{array}{c} 1\\ 0 \end{array} \end{array} \end{array}}} & \begin{array}{c} 0\\ \vdots \\ 0\\ \begin{array}{c} 0\\ 0 \end{array} \end{array} \end{array}\right]\cdot \theta_{j,GRN}\leq \left[\begin{array}{c} 0\\ 0\\ \vdots \\ \begin{array}{c} 0\\ 0 \end{array} \end{array}\right]$$

It is noted that the constraint we give in (10) could guarantee $d_{ju}$, parameters of miRNA, to be positive. That is $d_{ju}\geq 0$ for $u=1,2,\ldots ,U_{j}$ in the Equation (2).

### 4.5. System Order Detection Scheme for Obtaining Real GEN of Early and Later Stage AD

After performing the system identification for GENs in early and later stage AD, there still exist lots of false positives in the GENs. These false-positive interactions and regulations might be caused by various experimental conditions and noises. Therefore, we compute AIC to help us find the real system order. According to Akaike’s theory, the smallest AIC leads us to get the most accurate model [106]. In other words, the value of AIC achieves the minimum meaning that the detected system order approaches to the real system order. Moreover, the insignificant interactions and regulations remain inside the GENs of early and later stage AD would be pruned away.

The AIC value for the *i*th protein is defined as below:

(11) $$AIC_{i}\left(M_{i}\right)=\log\left(\frac{1}{N}{\left(P_{i}-\Phi_{i,PPIN}\cdot {\hat{\theta }}_{i,PPIN}\right)}^{T}\cdot \left(P_{i}-\Phi_{i,PPIN}\cdot {\hat{\theta }}_{i,PPIN}\right)\right)+\frac{2\left(M_{i}+1\right)}{N}$$

where ${\hat{\theta }}_{i,PPIN}$ denotes the estimated interaction parameter vector, which can be obtained by solving the Equation (6); $\frac{1}{N}{\left(P_{i}-\Phi_{i,PPIN}\cdot {\hat{\theta }}_{i,PPIN}\right)}^{T}\cdot \left(P_{i}-\Phi_{i,PPIN}\cdot {\hat{\theta }}_{i,PPIN}\right)$ indicates the estimated residual error. The lower the system interaction order is, the higher the corresponding estimated residual error has. In other words, there is a trade-off between system order and the estimated residual error. Our goal is to find system order $M_{i}^{*}$ of the *i*th protein, which minimize $AIC_{i}\left(M_{i}\right)$ in the Equation (11). By doing so, the insignificant PPI, which are out of the $M_{i}^{*}$, would be considered as false positives and be eliminated.

Similarly, to prune false-positive regulations in the GRN, the AIC value of the *j*th gene is given

(12) $$AIC_{j}\left(S_{j},T_{j},U_{j}\right)=\log\left(\frac{1}{N}{\left(G_{j}-\Phi_{j,GRN}\cdot {\hat{\theta }}_{j,GRN}\right)}^{T}\cdot \left(G_{j}-\Phi_{j,GRN}\cdot {\hat{\theta }}_{j,GRN}\right)\right)\;+\frac{2\left(S_{j}+T_{j}+U_{j}+1\right)}{N}$$

where ${\hat{\theta }}_{j,GRN}$ can be obtained by solving the Equation (10). $\frac{1}{N}{\left(G_{j}-\Phi_{j,GRN}\cdot {\hat{\theta }}_{j,GRN}\right)}^{T}\cdot \left(G_{j}-\Phi_{j,GRN}\cdot {\hat{\theta }}_{j,GRN}\right)$ is the estimated residual error. We aim to find $S_{j}^{*}$, $T_{j}^{*}$, and $U_{j}^{*}$ of the *j*th gene making $AIC_{j}\left(S_{j},T_{j},U_{j}\right)$ achieves the minimum. For the *j*th gene, the insignificant gene regulations, which are out of $S_{j}^{*}$, $T_{j}^{*}$, and $U_{j}^{*}$, can be regarded as false positives and be pruned away from the GRN. We conduct the same method to find out the real system order for the miRNA mode and lncRNA model. The details are shown in Supplementary Materials File.

### 4.6. PNP Method to Extract the Core GEN from Real GEN for Early and Later Stage AD

Despite possessing the real GENs, they are still too complicated for this study to investigate the pathogenic mechanism of AD. In order to investigate the pathogenic mechanisms and identify essential biomarkers for early and later stage AD, we have to extract the core GENs from the corresponding real GENs. The PNP method is a network projection approach based on the principal singular values. Before using it, we have to construct a network matrix Δ, which contains all the estimated interaction and regulation parameters in the real GEN as follows:

(13) $$\Delta =\left[\begin{array}{c} \begin{array}{ccccccccc} {\hat{a}}_{11} & {\hat{a}}_{12} & \cdots & {\hat{a}}_{1m} & \cdots & {\hat{a}}_{1M} & 0 & \cdots & 0\\ \vdots & \cdots & \cdots & {\hat{a}}_{im} & \cdots & \vdots & \vdots & 0 & \vdots \\ {\hat{a}}_{I1} & {\hat{a}}_{I2} & \cdots & {\hat{a}}_{Im} & \cdots & {\hat{a}}_{IM} & 0 & \cdots & 0\\ {\hat{b}}_{11} & \cdots & {\hat{b}}_{1s} & {\hat{c}}_{11} & \cdots & {\hat{c}}_{1T} & -{\hat{d}}_{11} & \cdots & -{\hat{d}}_{1U}\\ \vdots & {\hat{b}}_{js} & \vdots & \vdots & {\hat{c}}_{jt} & \vdots & \vdots & -{\hat{d}}_{ju} & \vdots \\ {\hat{b}}_{J1} & \cdots & {\hat{b}}_{JS} & {\hat{c}}_{J1} & \cdots & {\hat{c}}_{JT} & -{\hat{d}}_{J1} & \cdots & -{\hat{d}}_{JU}\\ {\hat{e}}_{11} & \cdots & {\hat{e}}_{1S} & {\hat{f}}_{11} & \cdots & {\hat{f}}_{1T} & -{\hat{h}}_{11} & \cdots & -{\hat{h}}_{1U}\\ \vdots & {\hat{e}}_{ks} & \vdots & \vdots & {\hat{f}}_{kt} & \vdots & \vdots & -{\hat{h}}_{ku} & \vdots \\ {\hat{e}}_{K1} & \cdots & {\hat{e}}_{KS} & {\hat{f}}_{K1} & \cdots & {\hat{f}}_{KT} & -{\hat{h}}_{K1} & \cdots & -{\hat{h}}_{KU}\\ {\hat{q}}_{11} & \cdots & {\hat{q}}_{1S} & {\hat{v}}_{11} & \cdots & {\hat{v}}_{1T} & -{\hat{w}}_{11} & \cdots & -{\hat{w}}_{1U}\\ \vdots & {\hat{q}}_{ls} & \vdots & \vdots & {\hat{v}}_{lt} & \vdots & \vdots & -{\hat{w}}_{lu} & \vdots \\ {\hat{q}}_{L1} & \cdots & {\hat{q}}_{LS} & {\hat{v}}_{L1} & \cdots & {\hat{v}}_{LT} & -{\hat{w}}_{L1} & \cdots & -{\hat{w}}_{LU} \end{array} \end{array}\right]\in {ℜ}^{\left(I^{*}+J^{*}+K^{*}+L^{*}\right)\times \left(S^{*}+T^{*}+U^{*}\right)}$$

where ${\hat{a}}_{im}$ is obtained in ${\hat{\theta }}_{i,PPIN}$ by solving the parameter estimation problem in (6) and pruning false-positives through AIC method in (11); ${\hat{b}}_{js}$, ${\hat{c}}_{jt}$ and $-{\hat{d}}_{ju}$ are obtained in ${\hat{\theta }}_{j,GRN}$ by solving the parameter estimation problem in (10) and pruning false-positives by AIC method in (12); ${\hat{e}}_{ks}$, ${\hat{f}}_{kt}$ and $-{\hat{h}}_{ku}$ are obtained in ${\hat{\theta }}_{k,LRN}$ by solving the parameter estimation problem in (s6) and pruning false-positives by AIC method in (s11); ${\hat{q}}_{ls}$, ${\hat{v}}_{lt}$ and $-{\hat{w}}_{lu}$ are obtained in ${\hat{\theta }}_{l,MRN}$ by solving the parameter estimation problem in (s10) and pruning false-positives by AIC method in (s12). The matrix Δ is padded with zeroes when the parameters are lacking or being pruned.

Extracting the core GEN, we apply PNP method to find principal components, which could reflect 85% of the original network. The network matrix, Δ, can be represented in the following form based on the singular value decomposition.

(14) $$\Delta =\Pi \times \Sigma \times \Psi^{T}$$

where $\Pi \in {ℜ}^{\left(I^{*}+J^{*}+K^{*}+L^{*}\right)\times \left(I^{*}+J^{*}+K^{*}+L^{*}\right)}$, $\Psi \in {ℜ}^{\left(S^{*}+T^{*}+U^{*}\right)\times \left(S^{*}+T^{*}+U^{*}\right)}$; and $\Sigma =diag\left(\sigma_{1},\ldots ,\sigma_{m},\ldots ,\sigma_{S^{*}+T^{*}+U^{*}}\right)\in {ℜ}^{\left(I^{*}+J^{*}+K^{*}+L^{*}\right)\times \left(S^{*}+T^{*}+U^{*}\right)}$. The components in the diagonal of Σ are the singular values of Δ. They are arranged in the descending order ($\sigma_{1}\geq \ldots \geq \sigma_{S^{*}+T^{*}+U^{*}}$). The eigen expression fraction $E_{m}$ is defined as below:

(15) $$E_{m}=\frac{\sigma_{m}^{2}}{\overset{S^{*}+T^{*}+U^{*}}{\underset{m=1}{\sum }}\sigma_{m}^{2}}$$

We select the minimum M such that $\overset{M}{\underset{m=1}{\sum }}E_{m}\geq 0.85$ so that the top M principal singular vectors can include 85% information of the real GEN in terms of energy point of view. Furthermore, we project the network matrix Δ to the top M singular vectors of Ψ The inner product is shown as below:

(16) $$B_{\Psi }\left(\omega ,m\right)=\delta_{\omega ,:}\times \psi^{T}{}_{:,m},\;for\;\omega =1,\ldots ,I^{*}+J^{*}+K^{*}+L^{*}\;and\;m=1,\ldots ,M$$

where $\delta_{\omega ,:}$ and $\psi_{:,b}^{T}$ represent the ωth row vector of Δ and denotes the *m*th column of $\Psi^{T}$, respectively. Afterwards, we give a description of the 2-norm projection value to each node, including proteins, genes, lncRNAs and miRNAs in the real GEN from the top *M* significant singular vector as below:

(17) $$D_{\Psi }\left(\omega \right)={\left[\overset{M}{\underset{m=1}{\sum }}{\left[B_{\Psi }\left(\omega ,m\right)\right]}^{2}\right]}^{\frac{1}{2}}\;,\;\mathrm{for}\;\omega =1,\ldots ,I^{*}+J^{*}+K^{*}+L^{*}$$

The larger the projection value $D_{\Psi }\left(\omega \right)$ is, the higher priority it has. In other words, the node with higher projection value would be selected to construct the core GENs for early and later stage AD, respectively. Compared to the real GENs in Figure S1, the core GENs own less nodes and edges as shown in Figure 2. This phenomenon reflects how the PNP method works.

## Figures and Tables

**Table 1:** The multiple-molecule drug for the early-stage AD.

Drug	Description	
Duloxetime	The number of targets	8
	Targets to be inhibited	ST13, IL-1R, CAMK1, SLC1A1, AQP4, TAB2
	Targets to be enhanced	GABRG2, APBA2
Sulindac	The number of targets	7
	Targets to be inhibited	ST13, IL-1R, CAMK1, SLC1A1, AQP4
	Targets to be enhanced	GABRG2, APBA2
Avagacestat	The number of targets	6
	Targets to be inhibited	ST13, IL-1R, CAMK1, SLC1A1, TAB2
	Targets to be enhanced	GABRG2

**Table 2:** The multiple-molecule drug for the later stage AD.

Drug	Description	
Trazodone	The number of targets	8
	Targets to be inhibited	EPHB2, EGFR, ARRB1, TNFR, FADD
	Targets to be enhanced	CDK5, AIG1, APC
Tideglusib	The number of targets	7
	Targets to be inhibited	EPHB2, EGFR, ARRB1, TNFR, FADD
	Targets to be enhanced	CDK5, APC
Epothilone D	The number of targets	7
	Targets to be inhibited	EPHB2, EGFR, ARRB1, TNFR, FADD
	Targets to be enhanced	CDK5, AIG1

## Data Availability

The Alzheimer’s microarray data comes from GSE84422 (https://www.ncbi.nlm.nih.gov/geo/query/acc.cgi?acc=GSE84422).

## Supplementary Materials

The following are available online at https://www.mdpi.com/article/10.3390/ijms222011280/s1.
