# Supplementary material for: Investigating Pathogenetic Mechanisms of Alzheimer’s Disease by Systems Biology Approaches for Drug Discovery
Source: Int J Mol Sci. 2021 Oct 19;22(20):11280. doi: 10.3390/ijms222011280 (PMC8540696; doi:10.3390/ijms222011280)
Supplement: Supplementary file 1 [file ijms-22-11280-s001.zip › ijms-1420918-supplementary.pdf]

Supplementary Materials

1.1. Systems modeling for the candidate GEN

In gene regulatory network, lncRNAs play a critical role to affect the downstream and upstream regulation of genes. The expression level of an lncRNA is regulated by its regulatory TFs/proteins, lncRNAs and miRNAs. The network is further defined as lncRNA regulatory sub-network (LRN). The lncRNA regulatory equation is shown as below:

$$x_k[n] = \sum_{s=1}^{S_k} e_{ks} p_s[n] + \sum_{t=1}^{T_k} f_{kt} x_t[n] - \sum_{u=1}^{U_k} h_{ku} x_k[n] r_u[n] + \beta_{k,LRN} + \varepsilon_{k,LRN}[n], \quad \text{(s1)}$$
$$for\ k = 1,...,K,\ n = 1,...N,\ h_{ku} > 0$$

where $x_k[n]$ is the expression level of the *k*th lncRNA; $p_s[n]$, $x_t[n]$ and $r_u[n]$ indicate the expression levels of regulatory TFs/proteins, lncRNAs and the miRNAs, respectively; e_{ks} denotes the transcription regulatory ability of the *s*th TF to the *k*th lncRNA; f_{kt} represents the transcription regulatory ability of the *t*th lncRNA to the *k*th lncRNA; h_{ku} is the post-transcriptional regulatory ability of the *u*th miRNA to inhibit the *k*th lncRNA; S_k denotes the total number of TFs binding to the *k*th lncRNA; T_k indicates the total number of lncRNAs binding to the *k*th lncRNA; U_k denotes the total number of miRNAs inhibiting the *k*th lncRNA; $\beta_{k,LRN}$ stands for the basal level to the *k*th lncRNA; $\varepsilon_{k,LRN}[n]$ shows the stochastic noise coming from the modeling residue and fluctuation in the *k*th lncRNA; *K* and *N* is the number of lncRNAs and patient samples, respectively.

In the same way, to express the interactions among miRNAs, TFs/proteins and lncRNAs, we construct a candidate miRNA regulatory sub-network (MRN). MiRNA regulatory equation is shown in the following:

$$r_l[n] = \sum_{s=1}^{S_l} q_{ls} p_s[n] + \sum_{t=1}^{T_l} v_{lt} x_t[n] - \sum_{u=1}^{U_l} w_{lu} r_l[n] r_u[n] + \beta_{l,MRN} + \varepsilon_{l,MRN}[n],$$
$$for\ l = 1,...,L,\ n = 1,...,N,\ w_{lu} > 0$$

(s2)

where $r_l[n]$ is the expression level of the *l*th miRNA; $p_s[n]$, $x_t[n]$ and $r_u[n]$ represent the expression levels of regulatory TFs/proteins, lncRNAs and miRNAs, respectively; q_{ls} indicates the transcription regulatory ability of the *s*th TF to the *l*th miRNA; v_{lt} shows the transcription regulatory ability of the *t*th lncRNA to the *l*th miRNA; w_{lu} is the post-transcriptional regulatory ability of the *u*th miRNA to inhibit the *l*th miRNA; S_l denotes the total number of TFs binding to the *l*th miRNA; T_l represents the total number of lncRNAs binding to the *l*th

miRNA; U_l is the total number of miRNAs inhibiting the lth miRNA; $\beta_{l,MRN}$ demonstrates the basal level of the lth miRNA; $\varepsilon_{l,MRN}[n]$ shows the stochastic noise coming from the modeling residue and fluctuation in the lth miRNA; L and N are the number of miRNAs and patient samples, respectively.

1.2. Systems identification to the candidate GEN via microarray data of early and later stage AD

Assisted with the corresponding microarray data, we apply systems identification to estimate interactive and regulatory parameters. The equation (s1) can be rewritten in the following:

$$x_k[n] = \begin{bmatrix} p_1[n] & \cdots & p_{S_k}[n] & x_1[n] & \cdots & x_{T_k}[n] & r_1[n] & \cdots & r_{U_k}[n] & 1 \end{bmatrix} \cdot \begin{bmatrix} e_{k1} \\ \vdots \\ e_{kS_k} \\ f_{k1} \\ \vdots \\ f_{kT_k} \\ -h_{k1} \\ \vdots \\ -h_{kU_k} \\ \beta_{k,LRN} \end{bmatrix} + \varepsilon_{k,LRN}[n] \equiv \phi^T_{k,LRN}[n] \cdot \theta_{k,LRN} + \varepsilon_{k,LRN}[n], \text{ for } k = 1,\dots,K$$

(s3)

where $\phi^T_{k,LRN}[n]$ is the regression vector, which can obtain from the microarray data. Moreover, $\theta_{k,LRN}$ indicates unknown parameter vector that have to be estimated in the LRN. There are N samples in AD microarray data. The regression form could be extended as:

$$\begin{bmatrix} x_k[1] \\ \vdots \\ x_k[N] \end{bmatrix} = \begin{bmatrix} \phi^T_{k,LRN}[1] \\ \vdots \\ \phi^T_{k,LRN}[N] \end{bmatrix} \cdot \theta_{k,LRN} + \begin{bmatrix} \varepsilon_{k,LRN}[1] \\ \vdots \\ \varepsilon_{k,LRN}[N] \end{bmatrix}, \text{ for } k = 1,\dots,K$$

(s4)

which could be simply described as

$$X_k = \Phi_{k,LRN} \cdot \theta_{k,LRN} + \mathrm{E}_{k,LRN} \quad \text{(s5)}$$

where

$$X_k = \begin{bmatrix} x_k[1] \\ \vdots \\ x_k[N] \end{bmatrix}, \ \Phi_{k,LRN} = \begin{bmatrix} \phi^T_{k,LRN}[1] \\ \vdots \\ \phi^T_{k,LRN}[N] \end{bmatrix}, \ \mathrm{E}_{k,LRN} = \begin{bmatrix} \varepsilon_{k,LRN}[1] \\ \vdots \\ \varepsilon_{k,LRN}[N] \end{bmatrix}$$

Therefore, by solving the following constrained linear least-squares parameter estimation problem in (s6), we could have the estimated regulatory parameters in the vector $\theta_{k,LRN}$.

$$\hat{\theta}_{k,LRN} = \arg\min_{\theta_{k,LRN}} \frac{1}{2}\left\|\Phi_{k,LRN} \cdot \theta_{k,LRN} - X_k\right\|_2^2$$

$$\text{subject to} \begin{bmatrix} \overbrace{0 \quad \cdots \quad 0}^{S_k+T_k} & \overbrace{1 \quad \cdots \quad 0 \quad 0}^{U_k} & 0 \\ \vdots \quad \vdots \quad \vdots & \vdots \quad \ddots \quad \vdots \quad \vdots & \vdots \\ 0 \quad \cdots \quad 0 & 0 \quad \cdots \quad 1 \quad 0 & 0 \\ 0 \quad \cdots \quad 0 & 0 \quad \cdots \quad 0 \quad 1 & 0 \\ 0 \quad \cdots \quad 0 & 0 \quad \cdots \quad 0 \quad 0 & 0 \end{bmatrix} \cdot \theta_{k,LRN} \leq \begin{bmatrix} 0 \\ 0 \\ \vdots \\ 0 \\ 0 \end{bmatrix} \quad \text{(s6)}$$

According to the above constraint, the lncRNA regulatory parameters, h_{ku}, are guaranteed to be positive by the inequality equation in (s6). That is $h_{ku} \geq 0$ for $u = 1, 2, ..., U_k$ in the equation (s1).

Similarly, the miRNA regulatory equation in (s2) could be rewritten as below:

$$r_l[n] = \begin{bmatrix} p_1[n] & \cdots & p_{S_l}[n] & x_1[n] & \cdots & x_{T_l}[n] & r_1[n] & \cdots & r_{U_l}[n] & 1 \end{bmatrix} \cdot \begin{bmatrix} q_{l1} \\ \vdots \\ q_{lS_l} \\ v_{l1} \\ \vdots \\ v_{lT_l} \\ -w_{l1} \\ \vdots \\ -w_{lU_l} \\ \beta_{l,MRN} \end{bmatrix} + \varepsilon_{l,MRN}[n] \equiv \phi_{l,MRN}^T[n] \cdot \theta_{l,MRN} + \varepsilon_{l,MRN}[n], \text{ for } l = 1, ..., L \quad \text{(s7)}$$

where $\phi_{l,MRN}^T[n]$ shows the regression vector that could be obtained from the microarray data, and $\theta_{l,MRN}$ denotes the unknown parameter vector, which have to be estimated. Since there are N samples in AD microarray data, the equation (s7) is extended as below:

$$\begin{bmatrix} r_l[1] \\ \vdots \\ r_l[N] \end{bmatrix} = \begin{bmatrix} \phi_{l,MRN}^T[1] \\ \vdots \\ \phi_{l,MRN}^T[N] \end{bmatrix} \cdot \theta_{l,MRN} + \begin{bmatrix} \varepsilon_{l,MRN}[1] \\ \vdots \\ \varepsilon_{l,MRN}[N] \end{bmatrix}, \text{ for } l = 1, ..., L \quad \text{(s8)}$$

which could be simplified in the following:

$$R_l = \Phi_{l,MRN} \cdot \theta_{l,MRN} + \mathrm{E}_{l,MRN} \quad \text{(s9)}$$

where

$$R_l = \begin{bmatrix} r_l[1] \\ \vdots \\ r_l[N] \end{bmatrix},\ \Phi_{l,MRN} = \begin{bmatrix} \phi_{l,MRN}^T[1] \\ \vdots \\ \phi_{l,MRN}^T[N] \end{bmatrix},\ \mathrm{E}_{l,MRN} = \begin{bmatrix} \varepsilon_{l,MRN}[1] \\ \vdots \\ \varepsilon_{l,MRN}[N] \end{bmatrix}.$$

Then, the regulatory parameters in the vector $\theta_{l,MRN}$ are estimated by solving the constrained linear least-squares estimation problem in (s10).

$$\hat{\theta}_{l,MRN} = \min_{\theta_{l,MRN}} \frac{1}{2} \left\| \Phi_{l,MRN} \cdot \theta_{l,MRN} - R_l \right\|_2^2 \quad \text{(s10)}$$

$$\text{subject to} \begin{bmatrix} \overbrace{0 \ \cdots \ 0}^{S_l+T_l} & \overbrace{1 \ \cdots \ 0}^{U_l} & 0 & 0 \\ \vdots \ \vdots \ \vdots & \vdots \ \ddots \ \vdots & \vdots & \vdots \\ 0 \ \cdots \ 0 & 0 \ \cdots \ 1 & 0 & 0 \\ 0 \ \cdots \ 0 & 0 \ \cdots \ 0 & 1 & 0 \\ 0 \ \cdots \ 0 & 0 \ \cdots \ 0 & 0 & 0 \end{bmatrix} \cdot \theta_{l,MRN} \le \begin{bmatrix} 0 \\ 0 \\ \vdots \\ 0 \\ 0 \end{bmatrix}$$

By setting the constraint by the inequality equation in the equation (s10), we can ensure

the miRNA repression parameters, w_{lu}, to be positive. In other words, it ensures that $w_{lu} \ge 0$ for $u = 1, 2, ..., U_l$ in the equation (s2).

1.3. System order detection scheme for obtaining real GEN of early and later stage AD

The AIC for the *k*th lncRNA is defined as below:

$$AIC_k(S_k, T_k, U_k) = \log\left(\frac{1}{N} \left(X_k - \Phi_{k,LRN} \cdot \hat{\theta}_{k,LRN} \right)^T \cdot \left(X_k - \Phi_{k,LRN} \cdot \hat{\theta}_{k,LRN} \right) \right) + \frac{2(S_k + T_k + U_k + 1)}{N}$$

(s11)

where $\hat{\theta}_{k,LRN}$ is computed by solving the equation (s6). $\frac{1}{N}\left(X_k - \Phi_{k,LRN} \cdot \hat{\theta}_{k,LRN} \right)^T \cdot \left(X_k - \Phi_{k,LRN} \cdot \hat{\theta}_{k,LRN} \right)$ is the estimated residual error. To get the real system order, we have to find the optimal S_k^*, T_k^*, and U_k^* making $AIC_k(S_k^*, T_k^*, U_k^*)$ achieves the minimum. The insignificant regulations by the *k*th lncRNA would be eliminated.

In the same way, the AIC for the *l*th miRNA is defined in the following:

$$AIC_l(S_l, T_l, U_l) = \log\left(\frac{1}{N} \left(R_l - \Phi_{l,MRN} \cdot \hat{\theta}_{l,MRN} \right)^T \cdot \left(R_l - \Phi_{l,MRN} \cdot \hat{\theta}_{l,MRN} \right) \right) + \frac{2(S_l + T_l + U_l + 1)}{N}$$

(s12)

where $\hat{\theta}_{l,MRN}$ indicates the estimated parameter vector, which can be obtained from solving the equation (s10). $\frac{1}{N}\left(R_l - \Phi_{l,MRN} \cdot \hat{\theta}_{l,MRN}\right)^T \cdot \left(R_l - \Phi_{l,MRN} \cdot \hat{\theta}_{l,MRN}\right)$ is the estimated residual error. For the lth miRNA, we have to find the optimal S_l^*, T_l^*, and U_l^* leading $AIC_l(S_l^*, T_l^*, U_l^*)$ to be the minimum. Moreover, the insignificant regulations, which are out of S_l^*, T_l^*, and U_l^*, would be regarded as false positives and be pruned away from the LRN.

Table S1. The enriched pathway analysis of core GEN in early stage AD.

Pathway analysis		
**KEGG pathway**	**Numbers**	**p-value**
GABAergic synapse	15	$2.30*10^{-3}$
Neurotrophin signaling pathway	12	$1.20*10^{-4}$
Toll-like receptor signaling pathway	10	$4.50*10^{-3}$
MAPK signaling pathway	9	$6.20*10^{-2}$
Wnt signaling pathway	8	$3.70*10^{-3}$

Table S2. The enriched pathway analysis of core GEN in later stage AD.

Pathway analysis		
**KEGG pathway**	**Numbers**	**p-value**
Jak-STAT signaling pathway	17	$4.60*10^{-4}$
Apoptosis	13	$2.70*10^{-3}$
Neurotrophin signaling pathway	9	$3.30*10^{-3}$
TNF signaling pathway	7	$5.10*10^{-2}$
MAPK signaling pathway	6	$1.40*10^{-2}$

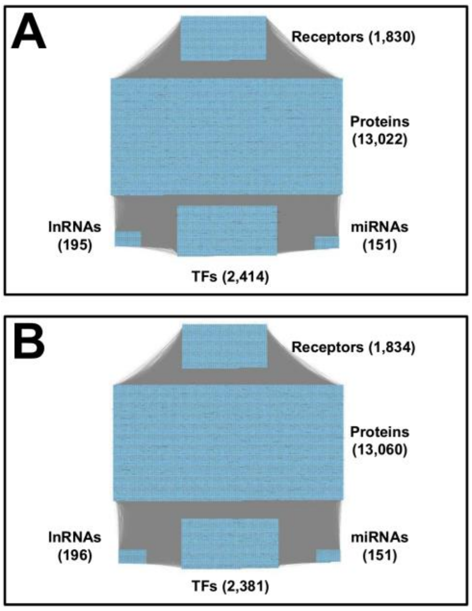

Figure S1. The identified real genome-wide GENs. Assisted with postmortem brain microarray data in **(A)** early stage AD and **(B)** later stage AD, we pruned the false positives from the candidate GEN to

obtain real GENs for early and later stage AD, respectively. The grey lines represent the interactions between groups, including receptors, proteins, miRNAs, TFs, and lncRNAs.
